# Acute coronary syndrome: which age group tends to delay call for help?

**DOI:** 10.1186/s43044-020-00124-7

**Published:** 2021-01-04

**Authors:** Ahmed Ayuna, Ayyaz Sultan

**Affiliations:** 1 Salford Royal NHS Foundation Trust, Salford, UK; 2 Royal Albert Edward Infirmary, WWL NHS Trust, Wigan, UK

**Keywords:** Acute coronary syndrome, Call for help, Delay presentation, STEMI, NSTE-ACS

## Abstract

**Background:**

Early diagnosis and treatment of ACS can reduce the risk of complications and death. Delay calling for help can increase morbidity and mortality. It is unclear which age group among patients with acute coronary syndrome tend to delay their call for help.

**Results:**

Our observational retrospective study showed that men and women in their 50s and 40s respectively tend to delay their call for help from symptoms onset. For the former, the mean time delays (590 ± 71.1 min), whereas for the latter it was (1084 ± 120.1 min). Moreover, these groups tend to have a longer time delay between symptoms onset and arrival at the hospital. Among deaths, we observed that the death rate was proportional to the time delay, which is not unexpected. Next step, we plan to perform a qualitative study in the form of questionnaires to target the individuals with a high risk of CVD within these age groups.

**Conclusion:**

Middle age group of both genders tend to delay their call for help when they experience symptoms of ACS; moreover, regardless of the age, the longer the delay, the higher the mortality rate. The results of this study gave us a better understanding of our local population and will pave the road for a well-structured teaching programme for them to minimise the time delay for calling for help.

## Background

Acute coronary syndrome (ACS) is a general term that includes a spectrum of two different main presentations: ST-elevation myocardial infarction (STEMI) and non-ST-elevation acute coronary syndrome (NSTE-ACS) [1]. ACS was the culprit behind 83,842 hospital admissions to the National Health Service (NHS) in England and Wales in 2014/2015 [2]. Data from the British Heart Foundation (BHF) suggested that around 530 individuals are hospitalised per day in the UK with acute myocardial infarction, whether STEMI or non-ST elevation myocardial infarction (NSTEMI) [3]. Interestingly, coronary artery disease was the single most common cause of premature death (age < 75 years) in 2012 in the UK [4]. It was the culprit behind about 15 % of men’s premature deaths in the UK and 8% in women [4]. It has been well-established that time to treatment in ACS is essential to reduce mortality [5]. Data from the USA in 2010 suggested that the average time delay from symptoms onset to hospital presentation was 2 h [6, 7]. Among the British population, it is unclear if there is a specific age group tend to delay their call for help from symptoms onset. The primary endpoint was to identify the population with the most extended delay from symptoms onset to call for help; the secondary endpoint was to detect the short-term outcome of delaying a call for help (7–30-day mortality during hospital admission). Next step, we plan to perform a qualitative study in the form of questionnaires to target the individuals with a high risk of cardiovascular disease (CVD) within this age group; this will help us in building a particular education program for them in collaboration with primary care providers.

## Methods

A cross-sectional observational retrospective study was carried out in a single centre (District General Hospital with onsite Cardiac catheterisation lab) in England, UK. Our centre is not a primary percutaneous coronary intervention (PPCI) centre. In our centre, the average annual admissions with chest pain from 01/01/2015 to 31/12/2017 were (1119.334 ± 117). This study analyse the data of the local Myocardial Ischemia National Audit Project (MINAP) which is part of the National Institute for Cardiovascular Outcomes Research (NICOR). A data governance framework by NICOR allows data sharing to research groups under the guidance of Data management group (DMG). Therefore, no ethical approval was required for this study. Authors analysed the data retrospectively for 1603 participants, and the follow-up was for 7 to 30 days during their stay in the hospital. Inclusion criteria included all the patients who were admitted to the hospital for the period from 01/04/2015 to 30/09/2017 and diagnosed with acute coronary syndrome; the ports of admissions were through the accident and emergency department, coronary care unit, and acute admission unit. The diagnosis of acute coronary syndrome was made based on the European Society of Cardiology (ESC) universal definition of myocardial infarction [1]. All-comer from both genders and different age groups were included to avoid selection bias. One thousand three hundred and seventy-seven (*n* = 1377) participants had NSTE-ACS, whereas 226 were admitted with STEMI or new LBBB before being transferred to a PPCI centre. Patients who had myocardial infarction during their stay in the hospital for another cause were excluded from the study. The main reason is to avoid confounding bias on the results of the secondary outcome.

Patients’ demographic data are mentioned in Table 1. The data includes the age, gender, ethnicity, previous history of ischemic heart disease whether myocardial infarction or angina, diabetes, hypertension and dyslipidaemia.

**Table 1:** It illustrates patients’ demographic data and past medical history

*N*	1603	100%
Male	919	57.33%
Female	684	42.66%
British white	1570	94%
Afro Caribbean	3	0.19%
Asian	8	0.50%
Mixed	4	0.24%
Not stated	16	1%
Other	8	0.50%
Previous AMI	509	31.75%
previous angina	672	42%
Hypertension	1023	63.81%
DM	466	29%
Dyslipidemia	886	55.27%

Descriptive statistics including mean (± the standard deviation) were computed in minutes. The primary analysis of the study has been descriptive, as it was an observational study. Continuous variables have been expressed as mean (± standard deviation), while categorical variables are presented as counts and percentage. Patients, males and females, were divided in different categories based on their age: < 20 years, 20–29, 30–39, 40–49, 50–59, 60–69, 70–79, 80–89, ≥ 90 years. We examined the time between symptoms onset and call for help, and the time from symptoms onset to hospital arrival to our centre. This information was documented by the ambulance crew and the clerking health care professionals in the admitting wards.

## Results

There were 1603 patients involved (*N* = 1603), mean age (70.4 ± 13.6) years, mode 77 years. Further, 57.33% were male (*n* = 919) and 42.67% were female (*n* = 684). Moreover, 97.94% were British white (*n* = 1570), 0.5% Asians (*n* = 8), 0.19% afro-Caribbean (*n* = 3), 0.25% mixed (*n* = 4) and 1.5% not stated (*n* = 24) (Table 1).

**Fig. 1:**
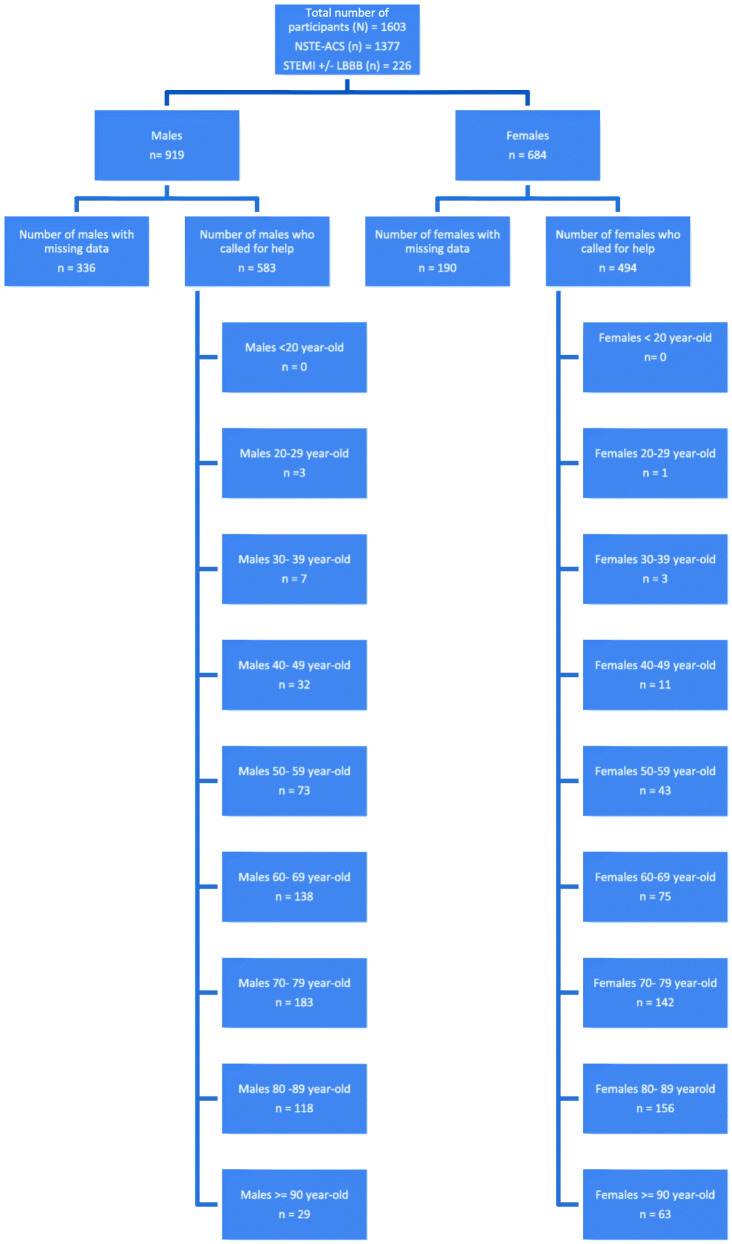
A flow chart demonstrates the number of participants from both gender and different age groups who called for help. It shows the number of participants with missing data for their time from symptoms onset to call for help

**Fig. 2:**
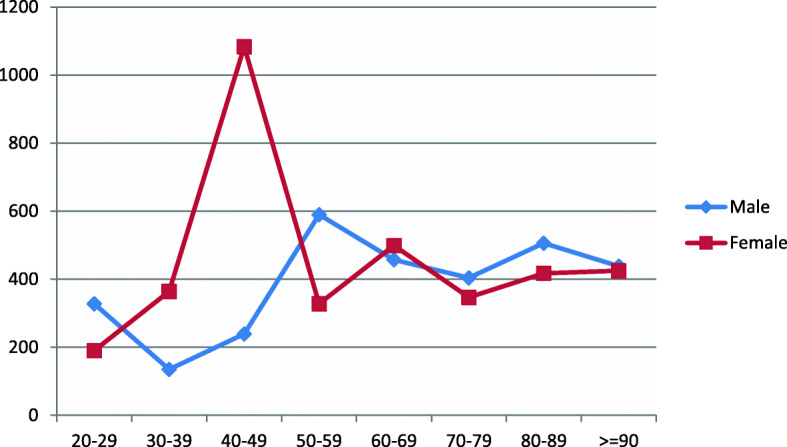
It demonstrates mean time delay in minutes from symptoms onset to ask for help for males and females

The total number for males called for help was 583/919; the data for the other 336/919 was missing; therefore, they were not included in this data analysis (Fig. 1). Men aged 50–59s tend to have the most prolonged mean time delay between symptoms onset and call for help which was (590 ± 71.1) min. Whereas males in their 30s tend to be quicker in asking for help; their figure was (135 ± 13.8) minutes. Men in their 80s, 90s and 60s have long delays before seeking medical advice; figures were (506 ± 61.1) min, (438 ± 55.2) min and (457 ± 54.8) min respectively (Table 2, Fig. 2).

**Table 2:** It demonstrates time delay between symptoms onset and call for help for male patients of different age groups

Age group	The mean time in minutes between onset of symptoms and call for help (min)	Standard deviation of the mean (SD)	Number of males
20–29	328	28.7	3 (0.51%)
30–39	135	13.8	7 (1.20%)
40–49	239	41.0	32 (5.40%)
50–59	590	71.1	73 (12.50%)
60–69	457	54.8	138 (23.67%)
70–79	404	43.8	183 (31.38%)
80–89	506	61.1	118 (20.24%)
≥ 90	438	55.2	29 (4.97%)

For female, the figures were slightly different; 494/684 called for help. The data for the other 190/684 was missing; therefore, it was not included in this data analysis. The most extended mean delays noticed for those aged 40–49 with (1084 ± 120.1) min. Generally, women tend to ask for help quicker than men (Table 3, Fig. 2).

**Table 3:** It illustrates time delay in minutes between symptoms onset and call for help for females of different age groups

Females Age groups	The mean time delay in minutes between symptoms onset and call for help	Standard deviation of the mean (SD)	Number and percentage of females
20–29	190	43.7	1 (0.20%)
30–39	364	83.2	3 (0.60%)
40–49	1083	120.1	11 (2.20%)
50–59	328	20.8	43 (8.70%)
60–69	499	54.7	75 (15.10%)
70–79	347	32.2	142 (28.74%)
80–89	417	44.1	156 (31.57%)
≥ 90	425	32.7	63 (12.75%)

**Fig. 3:**
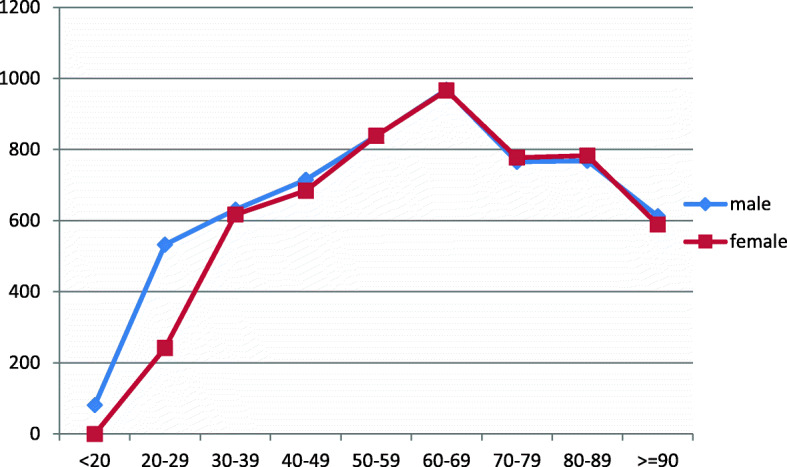
It illustrates mean time in minutes from symptoms onset to hospital arrival for Males and females of different age groups

As secondary findings, we noticed that the mean average of delays from symptoms onset to hospital arrival to our centre is generally almost the same for males and females (808 ± 21) min and (806 ± 28) min respectively. Interestingly, the maximum time delay was seen in individuals who are in their 60s; it was (968 ± 59.5) min for males (25.46%) and (966 ± 82.6) min for females (17.10%). Males and females in their 50s come after with figures of (839 ± 61.8) min and (839 ± 77.2) min respectively. Long delays were noticed in males in their 70s (26.76%), 80s (16.75%) and 90s (3.37%) at (765 ± 69.6) min, (768 ± 60.9) min and (613 ± 56.1) min respectively. For males younger than 20 years old, they had the minimum time delay; however, there was only one person who falls under this age-group. For females in their 70s (29.33%) and 80s (27.33%), they are both associated with long delays of (777 ± 65.4) min and (783 ± 66.7) min respectively. The figure for females ≥ 90 years old was (589 ± 29.3) min (Tables 4 and 5, Fig. 3).

**Table 4:** It demonstrates delays between onset of symptoms and arrival at the hospital in men of different age groups

Age of male patients	The mean time delay in minutes between symptoms onset and hospital admission (min)	Standard deviation of the mean (SD)	Number and percentage of male patients *n* (%)
< 20	81	12.1	1 (0.1%)
20–29	532	39.2	5 (0.5%)
30–39	631	63.6	14 1.5%)
40–49	715	92.1	73 (7.9%)
50–59	839	61.8	161 (17.5%)
60–69	968	59.5	234 (25.46%)
70–79	765	69.6	246 (26.76%)
80–89	768	60.9	154 (16.75%)
≥ 90	613	56.1	31 (3.37%)

**Table 5:** It demonstrates delays between onset of symptoms and arrival at the hospital in women of different age groups

Age in years	The mean time delay in minutes between symptoms onset and hospital admission (min)	Standard deviation of the mean (SD)	Number and percentages of females
< 20	0	0	0 (0%)
20–29	242	42.1	1 (0.14%)
30–39	617	47.2	6 (0.8%)
40–49	684	46.8	24 (3.5%)
50–59	839	77.2	78 (11.4%)
60–69	966	82.6	117 (17.1%)
70–79	777	65.4	200 (29.23%)
80–89	783	66.7	187 (27.33%)
≥ 90	589	29.3	71 (10.38%)

Less than one-tenth of the participants had sadly died during their hospital admission [8.5% (137/1603)]. From all deaths, 11/137 had STEMI, and the rest were NSTE-ACS (126/137). For a hundred and one of the total deaths (5 STEMI vs. 96 NSTE-ACS), there was > 121 min delay between symptoms onset and arrival to the hospital. The highest mortality was for people who were 70 years old and older (Tables 6 and 7).

**Table 6:** It shows the number of death and the associated mean time delay in minutes

Mean time delay in minutes	Number of deaths
30-120	36
121-360	49
>360	52

**Table 7:** It illustrates number and percentage of total deaths according to their age groups

Age group	*n* (%)
40–49	3 (2.18%)
50–59	4 (2.91%)
60–69	14(10.21%)
70–79	47 (35.03%)
80–89	40 (29.92%)
> 90	29 (21.16%)
Total number	137 (8.50%)
Total male deaths	73 (53.28%)
Total female deaths	64 (46.71%)

## Discussion

The findings from our study are interesting; it clearly showed that younger patients, regardless of their sex, tend to delay their call for help. The reasons behind these delays are not clear; however, Raczynski et al. argued that delays in calling for help could be related to patients cognitive and social variables [8]; for instance, they noticed that those who live alone tend to delay their call for help hoping the symptoms might resolve. There were shorter delays among those who were living with partners [8]. The differences between symptoms expectation and the actual experience of symptoms could be another reason behind the delays [9]. Sheifer and colleagues argued that the majority of the US population are aware of the association between chest pain and acute myocardial infarction; however, a significantly lesser number of people understand that acute coronary syndrome might present with less severe chest pain, atypical chest pain and even not a chest pain at all [10]. An American study reported that older people, females and minor ethnicity tend to delay their call for help [11]. Besides, they reported that socio-economic status was blamed for delay calling for help; the lower the socio-economic status, the longer the delays were noticed [11]. The differences could be related to the population’s education background and culture.

Furthermore, our study is smaller in sample size, single centre. Figures might change if a wide range of population from all over the country is included. The findings from our study are similar to the results reported by Ting and colleagues (2008). They reported longer time delays in patients younger than 60 years old compared with older people [7]. They attributed that to lack of patient awareness of the symptoms as they noticed a shorter delay in patients with previous MI and PCI. Our findings gave us a clear idea about our local population and will help us in our future planning. The number of deaths is proportional to time delay; the longer the delay, the higher the death rate, which is well known from previous studies and well-established [12]. Based on the results of this study, our next step will be a qualitative study, including a questionnaire to focus on these age groups during their hospital admission. The main target is to find out the reasons behind the delay. We are planning to design a specific education program to educate the local public, especially high-risk individuals about ACS, and the implications of late presentation. It will be in collaboration with primary care providers.

## Limitations of the study

It was a single-centre and observational study, not a randomised controlled trial. The data was collected from a single centre; therefore, the results might not be similar to another area inhabited by people of different demographic background. However, the authors are interested in introducing an educational programme to the area of interest. Furthermore, our centre is not a PPCI centre; therefore, some of the patients who are living in the area might be taken straight by ambulance to the PPCI centre without attending our hospital. Moreover, there was data missing with regard to time from symptoms onset to call for help from both gender; this has reduced the overall sample of participants. Finally, our centre is not a PPCI centre; therefore, one could argue that STEMI patients did not receive their appropriate care just after hospital arrival like their counterparts with NSTE-ACS which could affect the accuracy of the secondary outcomes. However, the number of deaths among STEMI patients was 11/137; this is much lower compared with NSTE-ACS (126/137); therefore, it is less likely to have a significant effect on the accuracy of the secondary outcomes of our study.

## Conclusion

Young people tend to delay their asking for help and have the most prolonged duration between symptoms onset and arrival to a hospital; this is true for both male and female. The death rate is higher for those in their 70s and older compared with younger patients. The results of this study gave us a better understanding of our local population and will pave the road for a well-structured teaching programme for them to minimise the time delay for calling for help.

## Data Availability

Not applicable.

## Abbreviations

ACS: Acute coronary syndrome
BHF: British heart foundation
CVD: Cardiovascular disease
ESC: European Society of Cardiology
LBBB: Left bundle branch block
MINAP: Myocardial infarction national audit project
NHS: National Health Service
NICOR: National Institute for Cardiovascular Outcomes Research
NSTE-ACS: Non-ST-elevation acute coronary syndrome
NSTEMI: Non-ST-elevation myocardial infarction
STEMI: ST-elevation myocardial infarction
UK: United Kingdom
US: United States
